# Observations on How People in Two Locations of the Plateau Département of Southeast Benin Perceive Entomophagy: A Study From West Africa

**DOI:** 10.3389/fnut.2021.637385

**Published:** 2021-02-24

**Authors:** Sampat Ghosh, Séverin Tchibozo, Euloge Lanmantchion, Victor Benno Meyer-Rochow, Chuleui Jung

**Affiliations:** ^1^Agriculture Science and Technology Research Institute, Andong National University, Andong, South Korea; ^2^Centre de Recherche pour la Gestion de la Biodiversité, Cotonou, Benin; ^3^Department of Ecology and Genetics, Oulu University, Oulu, Finland; ^4^Department of Plant Medicals, Andong National University, Andong, South Korea

**Keywords:** tradition, food system, insect as food, nutrition, indigenous traditional knowledge, sustainability

## Abstract

We surveyed the local populations of Kétou and Pobè in Southeast Benin through interviews and with the aid of a semi-structured questionnaire in order to understand how they currently perceive entomophagy, an age-old tradition in their communities. The study revealed that the majority of the population was familiar with the use of insects as food, and a sizable number of people were still interested in insect consumption. Gender differences were not apparent. Tradition or culture was identified as the most influential factor, followed by taste, as determinants for eating or rejecting insects. However, identifying the edible species and comparing practices how they were prepared for consumption, we found that the knowledge was not homogenous across the society of Benin, with differences depending on ethnicity, culture, respondent's age, and educational background. Awareness and promotion of food insects in the society should help to preserve the practice of entomophagy and in turn could lead to the provision of much needed nutritional supplements to the poorer and disadvantaged sections of the society.

## Introduction

The Republic of Benin, formerly known as Dahomey, is situated in West Africa between latitudes 6 and 13°N, and longitudes 0 and 4°E. The country is bordered by Togo to the west, Nigeria to the east, Burkina Faso to the northwest, and Niger to the northeast. In the surrounding countries and regions further to the south, a large number of insect species are considered as edible by the local inhabitants (1–6). However, compared with these countries, Benin stands out with a seemingly relatively sparse usage of insects as human food [see Table 1 in Kelemu et al. (4)].

Although there could be multiple reasons for this difference, a lack of field studies might well be involved, and insufficient knowledge of the causes why the people of Benin accept or reject edible insects species as human food could also have played a major role. There is, therefore, a need to question and to interview people. To date, this has not been done, and in a country like Benin with few roads and sometimes difficult access to villages and country areas, it is not exactly easy to organize. Moreover, not everyone is literate, and written questionnaires cannot be used as widely as would be desirable. The need to examine attitudes toward edible insects in the country is there, and the results of an inquiry such as ours, even if somewhat limited, could certainly be useful in comparisons with similar studies involving different regions and their residents in the future.

Food insect acceptance and consumption in Benin are likely to reflect the ethnic composition of the country and different cultural attitudes toward insects in general and food insects in particular. Approximately 42 ethnic communities are known from Benin. The Fon, also known as Dahomey, are the largest ethnic group and constitute ~39% of the total population of the country. Yoruba, i.e., 19% of the total population, reside mainly in the southeast and Dendi in the northern central part of the country, both having arrived during the twelfth to sixteenth centuries from regions that are now known as Nigeria and Mali, respectively. The Adja people of South Benin migrated from the River Mono to Benin during the twelfth and thirteenth centuries and at present constitute about 15% of the country's population. The northern part of the country is dominated by the Bariba people. The high levels and repeated waves of migration over different periods of time from a variety of regions inevitably led to interactions between different ethnicities. Adding to the wide spectrum of ecological and environmental conditions, it does not come as a surprise that cultural diversity is one of the characteristics of Benin and reflected in food habits and food taboos. To cite an example, 187 traditional leafy vegetables (TLVs) are used as food in Benin, but “the total number of TLVs used, highly varies across ethnic groups” (7).

Although suggested already in the 1970s by Meyer-Rochow (8) that insects could be an alternative sustainable food source to combat global food shortages, backed by the Food and Agriculture Organization of the United Nations (FAO) and WHO, the suggestion was not taken very seriously until a few decades ago. The insects' high nutritional value, their formidable food conversion efficiency, their need for less space and water, and their negligible greenhouse gas emission in comparison with conventional livestock were emphasized repeatedly (9, 10). By developing a food insect industry rather than collecting species in an uncontrolled manner from the wild, countries with a history of entomophagy could reap some benefits. Benin is one such country. Despite its apparently smaller use of edible insects than in the surrounding countries (4), there are several species of insects that have traditionally been accepted as food by the people of Benin (11–14). However, patterns of consumption and preparation processes vary among the different communities, and the concern remains how to preserve indigenous knowledge in the present era of globalization and the tendency of “westernization” (15).

The globalization of food brings two directions into conflict with each other: one is to keep the nutritional potential and ecological benefits of the insect source in mind (16), to proceed toward small-scale insect farming and to establish the legal framework necessary to reduce the pressure on the environment to generate more animal protein (17, 18). On the other hand, in some regions of the world, the availability of non-traditional, foreign-produced food items has become so dominant that consumers are ignoring traditional foods and see them as unfashionable and not sufficiently “modern” or “advanced” [(19), for edible insects: (20)]. This lack of appreciating and integrating tradition with new developments and food products often causes a one-sided dietary shift toward what is regarded as fashionable rather than time-honored (21, 22). Ultimately, the community is in danger of not only losing one important source of nutrition but also losing valuable indigenous knowledge associated with the practice of entomophagy. Benin is currently in the midst of a nutritional transition with an increasingly urban population (23).

The main food items for the people of southern Benin are known locally as *Akassa* (made of cornmeal), *Atassi* or *Watché* (made of bean and rice), and *Gari* (made up of grated cassava) (24). The cereal or cassava-based food is often limiting in amino acids. Inappropriate diets, promoting the onset of metabolic syndromes like cardiovascular disorders and diabetes (23, 25), often have a higher proportion of carbohydrates, less protein, and more saturated fats than foods considered healthy. According to the World Hunger Index, Benin is given a score of 22.4, which implies a serious level of hunger. Wasting, stunting, and micronutrient deficiencies persist among large segments, especially children and pregnant and lactating women, of the population^1^. Low-income regions in particular would benefit from the availability of a diverse diet that should include insects, as they are positively associated with micronutrient adequacy (26, 27).

This paper is a first and somewhat preliminary attempt to examine to what extent urban and rural people in one part of Benin appreciate the consumption of insects. Envisaging future studies of a similar nature, but then focusing on different regions and tribal people in Benin, it is hoped that this first investigation will be a stepping stone to explain why insects as a human food item seem less popular in Benin than in the surrounding countries and regions further south in Africa.

## Materials and Methods

### Study Area and Study Subjects

**Figure 1:**
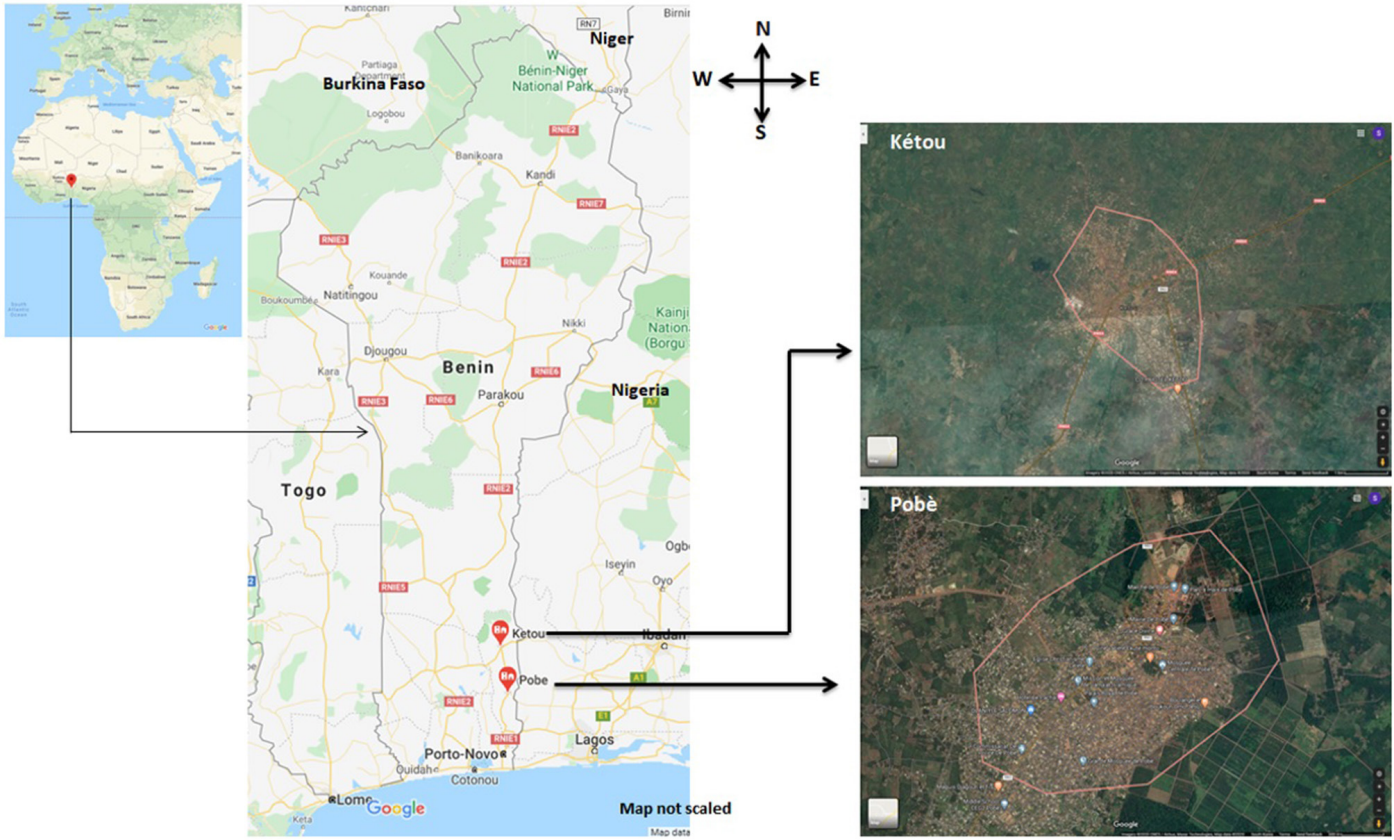
Survey area. The maps are adopted from Google Map.

We surveyed respective rural and urban populations of Kétou and Pobè in the southeastern part of Benin (Figure 1). Kétou is a small town of around 22,000 inhabitants in a rural area located in the Plateau Département de Benin. On the other hand, Pobè is a city, located in the same “département,” but with a total population of 123,740 according to the 2013 census. The climatic conditions of both places with annual temperature and relative humidity averages of 24°C and 91–92%, respectively, are the same.

### Survey and Data Collection

The survey's aim was to obtain some information on how inhabitants of the same “département,” but living in different towns, currently perceive entomophagy, an age-old tradition in their communities. Data from altogether 100 respondents were available. The rural people of Kétou (*n* = 50) that we interacted with belonged to seven ethnic communities, namely, Holli, Nagot, Fon, Yoruba, Adja, Goun, and Mahi. Kétou residents were mostly literate and could be interviewed with the help of a semi-structured questionnaire (Appendix A) translated into the common locally used French language. The semi-structured questionnaire and the methods of the interview were adopted from the study by Ghosh et al. (28). The emphasis of the questions was on testing familiarity with insects as a human food item, transmission of ancestral knowledge, and exploring the attitude of the respondents toward the use of insects as a normal part of the human diet. The filled-in questionnaires were collected immediately after the respondents had finished answering the questions or, in some cases, were collected within 48 h. It was inevitable that during contacts with the respondents, some conversation took place, and questions as to why this inquest was carried out had to be answered. However, only the written responses were processed.

On the other hand, due to the respondents' time constraints, impatience, and low literacy level, the 50 inhabitants of Pobè that had consented to be interviewed had to be asked to state their preferences for certain edible insect species verbally. This part of the survey was conducted with the help of photographs and available edible species from the market. The respondents were requested to indicate the edible species according to preference. Their answers were written down and later analyzed together with information on the respondents' background. We recorded information on the ethnic community to which a respondent belonged and the respondent's age, gender, profession, and educational level, but we did not include items like income and religious belief, as these are rather contentious issues in the society. It would have been nice to investigate in more detail also the roles that traditions and emotions play when people are faced with the choice to eat or not to eat insects. However, given the problems of accessibility and gaining the trust of the locals, such a study would have implied a more time-consuming and difficult data collection. As a first study on the topic, we believe that the results, even if limited in scope, will nevertheless be very useful when the project resumes.

The aims of the survey were explained to all rural as well as urban participants, and their consent to use their responses in a future report was obtained, provided no names of the respondents were mentioned. The study complied with the ethical guidelines of the institutions involved but regrettably contained unequal numbers of male and female participants. Traditionally, the women of rural folk are less inclined to speak openly and let the talking be done by the men. Women in an urban setting feel less inhibited and are more willing to answer questions.

### Identification of Edible Insects

Specimens of edible insects were collected and identified in the insect taxonomy laboratory of the Center de Recherche pour la Gestion de la Biodiversité (Cotonou, Benin), following standard key morphological characteristics.

### Data Analysis

The data obtained from the survey were then analyzed by SPSS (SPSS Inc., Chicago, IL, USA). Because of the relatively small number of respondents, Fisher's exact-tests was seen as superior to the X^2^-test (Chi-square test) and used to determine the independence of the responses associated with two nominal (categorical) variables like gender and knowledge system, gender and neophobicity, transmission of ancestral knowledge. It was felt that statistical analyses involving two tests could add support to the validity of our data, given that the number of the respondents was quite small. A Monte Carlo simulation (CI = 95% and sample size 10,000) was carried out wherever it was statistically possible. If the *p*-value was found to be ≤0.05 (CI = 95%), the null hypothesis was rejected.

## Results

### Socio-Cultural Characteristics of the Respondents

The demographic characteristics of the respondents are presented in Table 1. The highest number of respondents from rural area was Holli (36%), followed by Fon, accounting for 22%. The Holli people (a subgroup of the Nagot) live in Pobè, Kétou, and principally the region of the Lama Forest in southern Benin. Buffering the Lama Forest is a comparatively well-developed agro-biodiversity zone, in which people gather wild edible plants. Their diet is primarily based on maize, as also cited in the literature (29). Other ingredients include palm oil, salt, onion, garlic, tomatoes, and dried pepper. TLVs and fish, although not to a great extent, are also part of the food spectrum. For the Holli people of the Lama Forest, the contributions of wild edible plants regarding copper and iron were 13.9 and 4.6%, which are much below the estimated average requirement for women (29). Fruit consumption is also low, and meat is very rare, while eggs, milk, and dairy products are even less common. The Nagot people are dominant in the area of Sakété, Pobè, Kétou, Porto-Novo, and Savè.

**Table 1:** Demographic characteristics of the respondents from the Plateau Département of southeast Benin.

**Variables**	**Rural**		**Urban**	
	**Frequency**	**Percentage**	**Frequency**	**Percentage**
**Age (years)**				
<20	8	16	14	28
21–30	35	70	12	24
31–40	6	12	8	16
41–50	0	0	9	18
>50	1	2	7	14
**Gender**				
Males	42	84	24	48
Females	8	16	26	52
**Ethnic community**				
Holli	18	36	31	62
Nagot	4	8	17	34
Fon	11	22	0	0
Yoruba	6	12	0	0
Adja	2	4	0	0
Goun	7	14	0	0
Mahi	2	4	0	0
Bariba	0	0	1	2
Igbo	0	0	1	2
**Main profession**				
Farmer	2	4	17	34
Businessman	4	8	10	20
Craftsman	5	10	13	26
Student	35	70	10	20
Teacher	4	8	0	0

The respondents from the rural region of Kétou were aged between 16 and 51. Seventy percent (*n* = 35) of the respondents comprised individuals aged 21–30, followed by 16% of those younger than 21, and 12% of individuals 31–40 years of age. The informants who provided responses to questions on the perception of entomophagy unsurprisingly belonged predominantly to the younger generation. On the other hand, respondents capable of identifying edible insect species ranged from age 5 to 81.

### Perception of People Regarding Entomophagy

**Figure 2:**
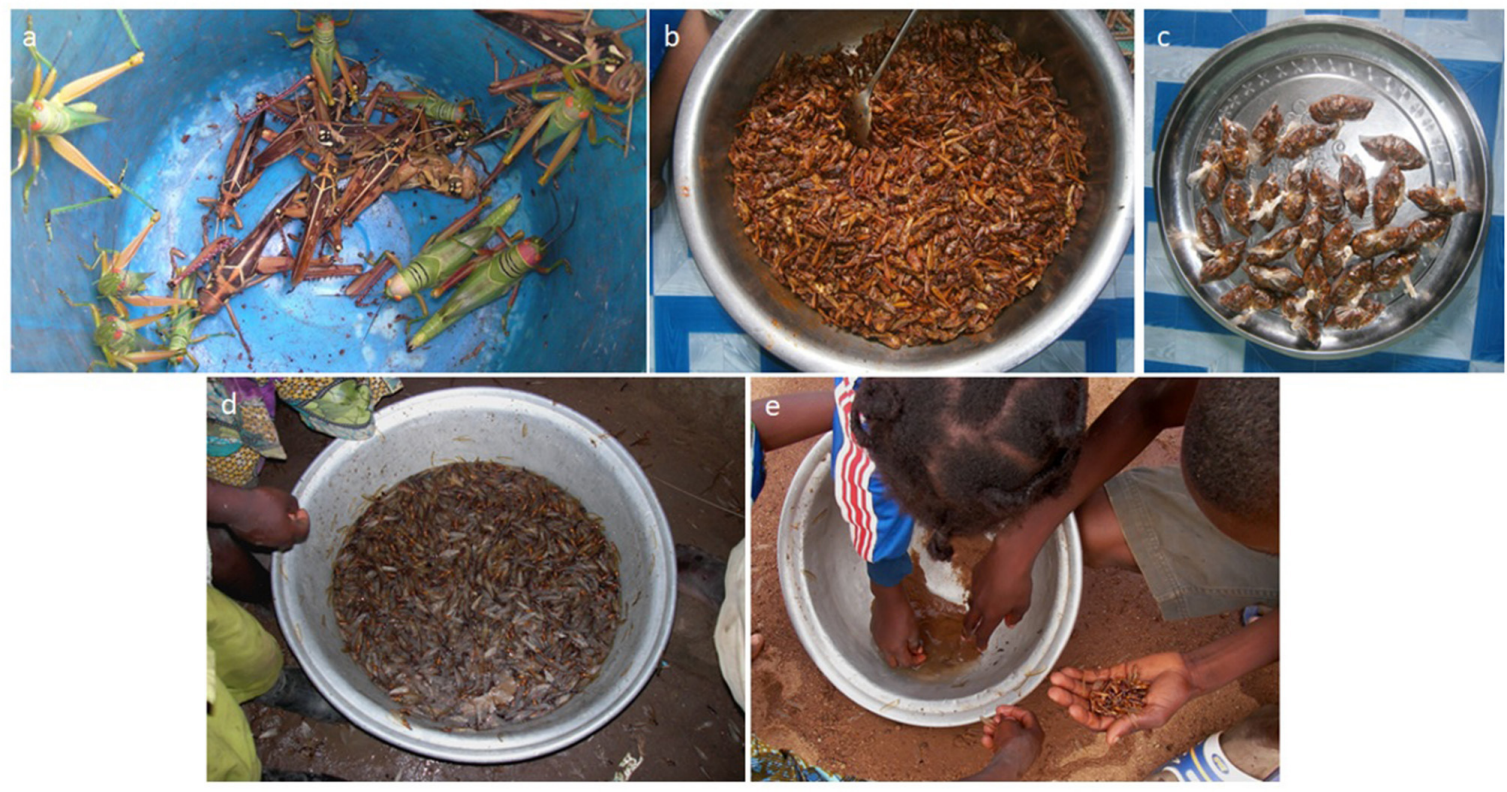
A selection of common edible insects consumed by people of the Plateau Département of Southern Benin. **(a)** Edible orthopteran species collected by children and adults of the rural community. **(b)** Processing of edible locusts by roasting. **(c)** Packaging of dewinged species for selling at the market (small-scale household industry). **(d)** Termites collected by children of the rural community. **(e)** Termites sorted by children of the rural community (photo credit: Séverin Tchibozo).

Results on the perception of the rural inhabitants of Kétou are represented in Table 2, and some commonly consumed edible insects are depicted in Figures 2a–e. The knowledge of eating insects, i.e., familiarity with entomophagy, was found to be independent of gender (Fisher's exact-test *p* = 1.000). In fact, 90.5% of the male and all female respondents of the interview indicated that they were well-aware of customary practice of eating certain insects. Not all Benin respondents had a desire to eat insects, but 45% of the male and 50% of the female respondents expressed an interest in consuming insects, and a gender difference was not apparent (Fisher's exact-test *p* = 1.000).

**Table 2:** Perception of rural people of Kétou in Southeast Benin regarding entomophagy.

**Variables**	**df**	**X**^****2****^		**Fisher's exact-test (2-sided)**
		**Value**	***p***	***p***
Gender × knowledge	1	0.828	0.363	1.000
Gender × interest	1	0.061	0.804	1.000
Gender × knowledge transmission from ancestor	1	1.691	0.193	0.261
Age group × knowledge transmission from ancestor*	3	1.384	0.709	0.900
Gender × feeling	1	0.050	0.823	1.000

* *Monte Carlo simulation (CI = 95%, sample no. = 10,000) was conducted for analysis of age group effect on the knowledge transmission, but was not significant (p = 0.900)*.

In Benin, 50% of the male and only 25% of female respondents declared that it was from their elders that they knew about the tradition of eating insects, while others stated that they were informed about insect-eating from different sources including friends, by seeing in the market the Nigerian people selling edible insects, by observing people collecting them, and by watching reports on insect eating on television, etc. There was no apparent gender bias with regard to the vertical transmission of indigenous knowledge (Fisher's exact-test *p* = 0.261).

In order to understand the nature of traditional knowledge transfer, we categorized the respondents into five different age groups: up to and including those aged 20, those aged 21–30, 31–40, 41–50, and above 50. Although we did not find any significant differences on the basis of age (Fisher's exact-test *p* = 0.900), we did find that respondents of the lower age group had received more information from their elders.

In our study, 78.6% of the male and 75% of the female respondents expressed positive feelings about eating insects, and no significant difference was found between the male and female respondents (Fisher's exact-test *p* = 1.000). Seventy-four percent of all respondents were willing to offer edible insects at social parties, while the remainder of 26% did not see this as a positive option.

In Benin, 80% of the respondents identified tradition as an influential factor in the acceptance of insects as a food item, while 70% identified the taste as the primary factor for eating insects, and a few respondents expressed nutrition as the primary factor of insect consumption. Except for tradition and an insect's taste, no respondent mentioned any other factor associated with choosing insects as food.

### Preferred Edible Insect Species in the Studied Urban Area

For the urban region, we identified as the most preferred edible insect species the larvae of the beetle *Rhynchophorus phoenicis* (known as *Woiwo* by members of the Nagot tribe and *Hli* by the Fon tribe), followed by crickets (e.g., *Brachytrupes* sp., known as *Hyrè* by the Nago and *Abosaklé* by the Fon) and termites of the species *Macrotermes falciger*, known as *Iba* by the Nago and *Toutou* by the Fon tribe. Other less common edible insect species included the grasshoppers *Zonocerus variegatus, Hieroglyphus africanus, Kraussaria angulifera, Ornithacris turbida*, and *Nomadacris septemfasciata*.

Edible insects are collected mainly for subsistence in times when the harvest is poor. They are washed, fried, and grilled or sometimes directly put into some spicy sauce and consumed by family members (Figures 2a–e). For the Nigerien diaspora, a sizable market exists in South Benin where Nigerian food insect enthusiasts import crickets and locusts from Niger and process them to be sold as a food item mainly to members of the Niger community and Nigerian expatriates living in the country (Figure 2c). In Pobè, too, some people collect insects to sell them in neighboring Nigeria. Processing of the orthopteran species involves frying pepper and chili powder added. The insects are usually not boiled, and the most popular way to prepare any insect for consumption by humans is to fry, roast, or grill them. Occasionally, insects may be eaten raw or added to a spicy sauce. Generally, both children and adults (men and women) of the family are engaged in collecting the edible species. However, a cricket (or any insect farming) industry, despite the use of *Brachytrupes* sp. as an edible species, does not yet exist in any part of the country.

## Discussion

### On the Perception of Entomophagy

Regrettably, the number of the people that could be interviewed was rather small, but given the total lack of information on insect consumption by the inhabitants of the southeastern region of Benin, this first survey of ours can still be considered useful, as it provides future studies to be conducted in this region with at least some data. One immediate result that is interesting in the context of familiarity with entomophagy is that by contrast to the present study, we found in an earlier investigation from East Africa that the majority of Ethiopian respondents (from Adama city) were completely unaware of the fact that insects could be regarded as human food (28). The contrasting results also held true in the context of gender and interest. Neophobia, which refers to phobia or fear of anything new, is an important parameter to assess a novel food (28, 30). Female Ethiopian as well as Korean respondents were more neophobic than men (28), but the reverse was found to be true in Benin. Although this is a far guess, as the Dahomey Kingdom had female military regiments, this could possibly have impacted the bravery of women in the country.

In most of the cases, indigenous knowledge plays a critical role in the utilization and conservation of natural resources. This dynamic body of knowledge is often transferred verbally generation after generation, and lack of scientific documentation often leads to the loss of the knowledge. Thus, we examined whether this knowledge of entomophagy remains among the different age groups and how this knowledge is transmitted. However, we did not have a sufficiently large enough database to allow us to draw a firm conclusion. In contrast, one study in Botswana revealed that older people were more knowledgeable and familiar with a wider range of edible species than the younger generation (31), which is in agreement with a survey on edible insects in Northeast India (32). The importance of traditional knowledge in the food culture in the context of entomophagy has been discussed in detail by Sogari et al. (33), who cited comparative accounts between western and eastern societies, while the factors influencing food choice mechanism, particularly in the context of edible insects, have been reviewed by Ghosh et al. (34). Very few respondents expressed nutrition as the factor guiding them in which species of insect to consume. In this context, the rich ethno-entomological wisdom of the Sub-Saharan Kalahari San people (35) and the sustainable use of insects as food in Sub-Saharan Africa (36) generally are remarkable, but more recent data on present-day attitudes toward entomophagy are not available.

Members of the surveyed populations in Benin were found to have positive feelings about entomophagy, a result that is at variance with findings of a previous study involving an Ethiopian population. A majority of the Ethiopian respondents expressed their unwillingness to accept insects as food and found insect consumption not to be “culturally superior” (28). Similarly, in a study conducted in Italy, the majority of the respondents stated that entomophagy would not be endorsed or supported by family and friends and that this negative opinion would represent a barrier to accepting insects as a novel food (37). Hedonic senses like taste, flavor, texture, and coloration generally play important roles in food selection, but one study with blindfolded and nose-clipped respondents showed that food insects could not be distinguished from non-insect food items by taste alone (38). In Benin, except for tradition and an insect's taste, no respondent mentioned any other factor associated with choosing insects as food. Taste, incidentally, was also found to be a dominant factor followed by nutrient content and environmental issues in the case of Korean and Ethiopian respondents (28).

Although this present study focused on the southeastern part of Benin, it has been noted that different species of insects are consumed by different ethnic communities in North and South Benin (11, 12). Overall, the consumption of insects in Northern Benin appears to be more common than in the southern part of the country, presumably because of different agro-climatic conditions and thus abundance and occurrence of different insects (14). However, diverse ethnic communities, traditional knowledge, presence of natural landscape, and less urbanization in the northern part would also contribute to the difference. Termites of the genus *Macrotermes*, the gryllid *Brachytrupes* sp., and grasshoppers of the genus *Hieroglyphus* are among the most common genera of edible insects in both North and South Benin, but nowadays, none of these species is consumed much.

### Possible Role of Insects in Improving Nutrition

We described in the previous introductory section that there is a gap existing between the desired nutritional level and the nutrition obtained from the local food. As innumerable scientific reports have demonstrated, the nutritional value of edible insects is no longer in doubt (39). The consumption of insects can enhance health, as most of the edible species contain high amounts of protein and minerals especially iron, calcium, and zinc, and less total fat but generally a higher proportion of unsaturated fatty acids, e.g., crickets (40, 41), honey bees (42–45), and termites (46, 47). However, as the finding of the present study has confirmed, acceptability of insects as food for humans does not solely depend on the nutritional potential of the resource but includes many other factors (34, 48–50). As with other nutritious but neglected traditional food items, edible insects are getting increasingly shunned in areas where they had been consumed for centuries but have received more and more attention by consumers in countries that are considered developed (31, 51). However, the consumer acceptance is rather a complex issue, and framework is required to innovate or develop proper strategy and promote edible insects (52).

### Further Prospects on Development of Edible Insects in Benin Context

Benin, with an increasing urban share of the population, is in the midst of a nutritional transition (25). Awareness and integration of traditional practices with modern trends may protect those cultural characteristics that are associated with the utilization of local resources including edible insects. However, we do not outrightly advocate the uncontrolled harvesting of edible insects from the wild. We suggest instead that insect farming systems receive the support they need in order to prosper. Herbivorous insects, feeding on plants in the wild, can accumulate several secondary metabolites with anti-nutritional properties that offer protection to plants but are usually not desired in edible species of insects (46, 53).

Farmed insects can be monitored with regard to the amount of anti-nutrients in their bodies (54). Having started barely 20 years ago, as of 2016, there have been 724 registered cricket farms in Korea (55, 56). Farming systems of a similar nature with the necessary and associated legal framework have been developed or are in the process of being set up in many countries of Asia, Europe, Africa, and North America (18, 57). Establishments such as these do not only ensure the systematic supply of products of high nutritional value, but they also generate employment opportunities, which enhance the local economy and serve the well-being of the society. However, at present, there are no insect farming or rearing facilities in Benin.

### Some Questions Arising From Our Survey

Based on our survey, safety turned out to be one of the important issues of concern. Traditionally, edible insects are harvested from wild areas by the local people, and they are aware of aposematic coloration that warns them of harmful and toxic individuals. But some insects, seemingly not dangerous in any way, may feed on a variety of herbs that contain allelochemicals like polyphenol and flavonoids, which may have anti-nutrient properties and build up in the insects' body (46, 53). Moreover, pathogenic microorganisms in the insects' gut, fat body, brain, or endocrine organs could affect human health negatively. Among pathogenic bacteria, *Salmonella* spp., *Escherichia coli, Staphylococcus aureus, Enterococcus faecalis, Enterococcus faecium, Aeromonas hydrophila, Bacillus cereus, Clostridium perfringens*, etc., may pose additional potential hazards to human health (58).

A reduction of moisture content would help to avoid microbial contamination and increase shelf life, but drying edible insects prior to their use is rarely practiced in Benin (11). Blanching, freeze-drying, oven drying, cold atmospheric pressure plasma treatment, etc., are methods employed for the reduction of microbial load of edible insects in modern food processing, but not in Benin (59). Suitable processing is also important for the extraction of desired compounds or groups of compounds like chitin, protein, and fat. Although such advanced processing techniques are not found in the surveyed area, techniques that the locals use like frying, grilling, and, on some occasions, boiling, are methods that will to some extent take care of pathogens and may reduce water-soluble anti-nutrient contents like trypsin inhibitor and oxalate etc. present in some insects, as these methods are found effective for some plant foods (60, 61). Scientifically monitored production systems and processing technology now available in many European and Asian countries still need to be developed for Benin (59). Proper maintenance of hygiene and sanitation of any insect farm to avoid contamination are of course as essential as conserving the wild species of interest and the knowledge associated with their roles in nature as a potential source of food and medicines.

## Conclusion

Most people of Benin are familiar with entomophagy, but the knowledge regarding edible insect species is not homogeneous across the population of the “Plateau Département” of southeastern Benin; noted differences depend on ethnicity, culture, age, etc. Awareness and promotion of food insects in the society should help to preserve the practice of entomophagy and in turn could lead to the provision of much needed nutritional supplements to the poorer and disadvantaged sections of the society. Spin-offs like the establishments of insect-rearing facilities could possibly improve the livelihood of some farmers and enhance the economic growth of certain localities.

## Data Availability Statement

The original contributions generated in the study are included in the article/Supplementary Material, further inquiries can be directed to the corresponding author/s.

## Author Contributions

SG: design, data curation, analysis, and writing manuscript. ST and EL: field data collection and editing manuscript. VBM-R: design and editing manuscript. CJ: funding, design, analysis and editing manuscript. All authors contributed to the article and approved the submitted version.

## Conflict of Interest

The authors declare that the research was conducted in the absence of any commercial or financial relationships that could be construed as a potential conflict of interest.

## Footnotes

^1^https://www.globalhungerindex.org/benin.html (accessed January 2, 2021).
