# Supplementary material for: Observations on How People in Two Locations of the Plateau Département of Southeast Benin Perceive Entomophagy: A Study From West Africa
Source: Front Nutr. 2021 Feb 24;8:637385. doi: 10.3389/fnut.2021.637385 (PMC7943480; doi:10.3389/fnut.2021.637385)
Supplement: Supplementary file 1 [file Table_1.DOCX]

Appendix-A

**Questionnaire about insect eating:**

Your age (Votre âge): ___Gender (Sexe): ______Work/Job (Travail): ____________

1. Are you informed about entomophagy (practice of eating insect as food)? (Êtes-vous informé de l'entomophagie (pratique de manger des insectes comme nourriture)?)
2. Yes. Please mention the source of your information

(Oui. Veuillez mentionner la source de vos informations)

______________________________

1. No (Non)
2. Little (sometimes I heard the term but not understood fully)

(Peu (parfois j'ai entendu le terme mais je ne l'ai pas bien compris))

1. Are you interested to eat insect food?

(Êtes-vous intéressé à manger de la nourriture à base d’insectes?)

1. No, not at all

(Non pas du tout)

1. Yes

(Oui)

1. Only if insect is used as ingredient (I don’t like to see an insect as my food)

(Seulement si l'insecte est utilisé comme ingrédient (je n'aime pas voir un insecte comme ma nourriture))

1. Have you ever heard from your ancestor about insect food?

(Avez-vous déjà entendu parler de votre ancêtre au sujet de la nourriture pour insectes?)

1. No

(Non)

1. Yes. Please name ____________________ (local name is okay)

(Oui. Veuillez nommer ____________________ (le nom local est correct))

1. I did not hear from my ancestor but from some other fellow.

(Je n'ai pas eu de nouvelles de mon ancêtre mais d'une autre personne.)

1. What kind of feeling do you have about entomophagy?

(Quel genre de sentiment ressentez-vous à propos de l'entomophagie?)

1. Positive

(Positif)

1. Negative

(Négatif)

1. I really don’t have any feeling

(Je n'ai vraiment aucun sentiment)

1. We have some food products in which we use insect as an ingredient. Do you like to taste?

(Nous avons certains produits alimentaires dans lesquels nous utilisons des insectes comme ingrédient. Aimez-vous goûter?)

1. No

(Non)

1. Yes

(Oui)

1. Let me see the texture/shape of food and taste

(Permettez-moi de voir la texture / la forme des aliments et le goût)

1. Would you like to offer any insect dish in your small personal function

(Souhaitez-vous offrir un plat à insectes dans votre petite fonction personnelle)

1. No. I can’t think of it

(Non, je ne peux pas y penser)

1. Yes

(Oui)

1. I can offer in some tea party

(Je peux offrir dans une partie de thé)

1. Do you have any question regarding ‘entomophagy’. Please feel free to ask us

(Avez-vous des questions concernant l’entomophagie? N'hésitez pas à nous demander)

_________________________________________________________________________________________________________________________________________________________________________________________________________________________________

Thank you very much for your sincere response. We assure you (Respondent) that your response will be used for academic research purpose only.

(Merci beaucoup pour votre sincère réponse. Nous vous assurons (répondant) que votre réponse ne sera utilisée qu'à des fins de recherche universitaire.)
